# Extrahepatic toxicity of acetaminophen: critical evaluation of the evidence and proposed mechanisms

**DOI:** 10.18053/jctres.03.201703.005

**Published:** 2017-11-18

**Authors:** Stefanie Kennon-McGill, Mitchell R. McGill

**Affiliations:** ^1^ Department of Environmental and Occupational Health, Fay W. Boozman College of Public Health, University of Arkansas for Medical Sciences, Little Rock, Arkansas, United States; ^2^ Department of Psychiatry, Washington University School of Medicine, St. Louis, Missouri, United States

**Keywords:** liver injury, kidney injury, endocrine disruptors, neurotoxicity, ototoxicity

## Abstract

Research on acetaminophen (APAP) toxicity over the last several decades has focused on the pathophysiology of liver injury, but increasingly attention is paid to other known and possible adverse effects. It has been known for decades that APAP causes acute kidney injury, but confusion exists regarding prevalence, and the mechanisms have not been well investigated. More recently, evidence for pulmonary, endocrine, neurological, and neurodevelopmental toxicity has been reported in a number of published experimental, clinical, and epidemiological studies, but the quality of those studies has varied. It is important to view those data critically due to implications for regulation and clinical practice. Here, we review evidence and proposed mechanisms for extrahepatic adverse effects of APAP and weigh weaknesses and strengths in the available data.

**Relevance for patients:**

APAP is one of the most commonly used drugs in the West. Although it is generally considered safe when used according to manufacturer recommendations, it has been known for decades that overdose can cause liver injury. Recent studies have suggested that APAP can damage cells in other organs as well, leading to calls for more and stricter regulations, which would limit use of this otherwise effective drug. It is especially important to view claims of developmental effects of antenatal APAP exposure with a critical eye because APAP is currently the only over-the-counter medication recommended for pregnant women to self-treat pain and fever.

## 1. Introduction

Acetaminophen (APAP; a.k.a. paracetamol) is one of the most commonly used drugs in the US [1] and throughout the West, but has a relatively low therapeutic index. The major target organ of APAP toxicity is the liver. In fact, APAP is the principal cause of acute liver failure (ALF) and related deaths in several countries [2]. The hepatotoxicity of APAP was first reported in the 1960s [3-5]. In the five decades since those initial reports, studies of APAP toxicity have focused almost exclusively on the prevalence and mechanisms of liver injury. Recently, however, attention has shifted toward other adverse effects. A large number of studies have reported neurological [6-14], pulmonary [15-21] and developmental toxicity [6,7,11,14,22] in both preclinical models and humans.

It is important to critically evaluate the evidence for toxic effects of any drug or other xenobiotic. Claims of toxicity can lead to changes in clinical practice or regulation that can affect patient care. Recently, concerns regarding liver injury caused by APAP have led the US FDA to reduce the maximum amount of APAP allowed in prescription formulations to 325 mg, and to recommend lower daily doses for over-the-counter use [23]. It is especially important to view claims of developmental and congenital effects of intrauterine APAP exposure with a critical eye because APAP is currently the most commonly used drug among pregnant women and for many years was the only analgesic considered safe for use during pregnancy [24,25], a perception that still exists among many clinicians and patients. An association between APAP use in pregnancy and disease in offspring could easily lead to changes in clinical practice, just as associations between NSAIDs and various adverse outcomes such as low birth weight, birth defects, and child mortality led the FDA to classify aspirin and others as category D for pregnancy, meaning that there is positive evidence for maternal fetal risk, and caused clinicians to recommend against their use [24].

The purpose of this review is to summarize studies of adverse extrahepatic effects of APAP and to evaluate the evidence for those effects. Animal studies, human studies and epidemiological reports are discussed. Special attention is given to the pathophysiological mechanisms that have been proposed to explain the phenotypic findings from those data. The review begins with what is known about the mechanisms of toxicity in the liver, and findings from other organs are discussed with reference to those well-known mechanisms. Overall, it is clear that APAP is toxic in other organs, but the quality of the evidence and mechanisms varies. In many cases, there is a paucity of mechanistic data, or the available mechanistic studies suffer from poor design. However, that does not necessarily invalidate observations of adverse effects. We strongly recommend that future investigations use only reliable in vivo models and doses that are relevant for the human context.

**Table 1.** Proposed extra-hepatic adverse effects of APAP

Toxicity	Evidence	Proposed mechanisms	Comments
Renal	Clinical and rodent studies	Protein binding, ɤ-glutamyl cycling	Strong human and rodent data
Pulmonary	Epidemiology, limited preclinical studies	GSH depletion, oxidative stress, neurogenic inflammation	Better study designs needed
Endocrine	Epidemiology, limited preclinical studies	Altered sex steroid metabolism, inhibition of prostaglandin synthesis	Conflicting human and experimental data
Ototoxicity	Case reports, limited preclinical studies	Oxidative stress, ER stress	Strong human data, conflicting experimental data
Neurobehavioral	Epidemiology, limited preclinical studies	Endocrine disruption, endocannabinoid signaling, direct neurotoxicity	Better study designs needed

## 2. Overview of APAP metabolism and hepatotoxicity

**Figure 1.**
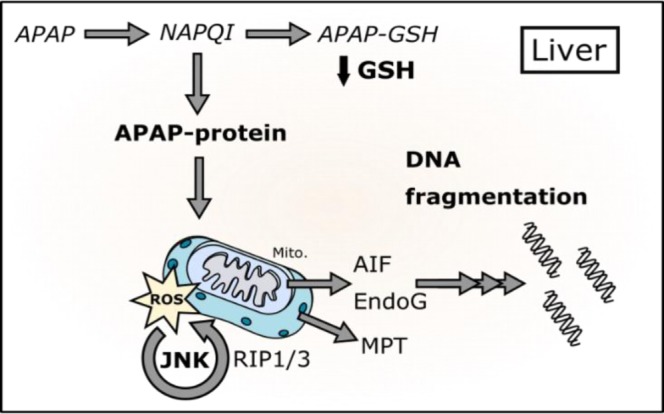
Pathophysiology of APAP-induced liver and kidney injury. Most of a dose of acetaminophen (APAP) is glucuronidated or sulfated in the liver and then excreted. A small percentage in both the liver and kidney is converted to the electrophilic intermediate N-acetyl-p-benzoquinone imine (NAPQI). NAPQI can be detoxified by reaction with glutathione (GSH), which depletes GSH stores. NAPQI can also bind to proteins, which leads to cell death. The mechanisms of cell death in the liver include mitochondrial oxidative stress, c-Jun N-terminal kinase (JNK) activation and nuclear DNA fragmentation (inset). In the kidney, GSH depletion is exacerbated by the GGT cycle, which enhances the nephrotoxicity.

Although several critical details are still missing, the metabolism and toxicity of APAP in the liver have been thoroughly investigated [26] (Figure 1). After therapeutic doses, approximately one-third is glucuronidated while another third is sulfated [26, 27]. Any remaining parent compound is converted by cytochrome P450 enzymes to an electrophilic intermediate, believed to be N-acetyl-p-benzoquinone imine (NAPQI) [28]. Binding of the reactive metabolite to proteins is known to be the initiating event in liver injury [29-32]. Binding to mitochondrial proteins appears to be particularly important. Changes in mitochondrial function and integrity are known to occur in the liver after APAP overdose in both mice and humans [15, 33 - 36]. Interestingly, the reactive metabolite of N-acetyl-p-aminophenol (AMAP), an isomer of APAP, binds much less to mitochondrial proteins in primary mouse hepatocytes (PMH) than the metabolite of APAP, and PMH are much less susceptible to the toxicity of AMAP than of APAP [37]. Furthermore, unlike PMH, AMAP treatment does result in mitochondrial protein adducts in primary human hepatocytes (PHH) [37], which are damaged by AMAP [37,38]. Finally, rats are less susceptible to APAP hepatotoxicity than mice and also have less mitochondrial protein binding after APAP overdose [39]. Together, those data strongly suggest that mitochondrial protein binding is critical.

Although it is not known exactly how it occurs, the mitochondrial protein binding seems to cause oxidative stress. The major reactive oxygen species (ROS) in APAP hepatotoxicity are superoxide (O2-) and peroxynitrite (ONOO-) [40], which form primarily within mitochondria and drive the injury [40-46]. Replenishment of glutathione by treatment with the precursor *N*-acetylcysteine (NAC) protects against APAP hepatotoxicity not only by scavenging the reactive metabolite of APAP, but also by reducing oxidative stress [47,48].

The initial oxidative stress after APAP overdose activates mitogen - activated protein kinases (MAPKs), including the cJun N-terminal kinases (Jnk) 1/2 [49,50] (Figure 1). The role of Jnk 1/2 is controversial. The Jnk 1/2 inhibitor SP600125 protects against APAP toxicity in mice in vivo and in both PMH and PHH [51,52]. Although some groups have also shown protection with knockdown or knockout of Jnk isoforms, particularly Jnk2 [51], others have failed to reproduce those results [52-55]. The discrepancy between different studies that utilized *Jnk2* deficient mice may be due to use of control animals from different substrains [56]. Interestingly, one recent study demonstrated that neither *Jnk 1* nor combined *Jnk 1/2* deficiency in the liver is protective against APAP hepatotoxicity [55]. In fact, *Jnk1/2* knockout appeared to worsen injury [55]. Furthermore, SP600125 protected in the double knockout mice [55]. The authors concluded that Jnk 1/2 is not part of the mechanism of toxicity and that SP600125 protects through off-target effects [55]. However, those results do not explain why other Jnk 1/2 inhibitors also protect against APAP [53,57]. Overall, the weight of the evidence favors a role for Jnk [58]. Once activated, Jnk 1/2 translocates to mitochondria [44,59], and it is thought that it enhances the mitochondrial oxidative stress [59,60]. Other kinases that have been shown to play a role in mice include the mixed lineage kinase 3 (Mlk3) [61] and the receptor interacting protein kinases (Ripk) 1 and 3 [62-64]; however, their exact mechanisms are unclear.

The mitochondrial permeability transition (MPT) is also a critical step in the mechanism of APAP-induced liver injury (Figure 1). MPT inhibitors and genetic deletion of MPT pore components protect against APAP hepatotoxicity both *in vitro* and *in vivo* [34,65-67]. The resulting mitochondrial swelling leads to lysis of mitochondria and release of mitochondrial contents [35,68,69]. Mitochondrial endonucleases, in particular, are liberated and translocate to nuclei where they cleave genomic DNA [69]. Although nuclear DNA fragmentation is widely considered a hallmark of apoptosis, oncotic necrosis is actually the major mode of cell death in the liver after APAP overdose. Studies in both humans and mice demonstrate that apoptosis has, at most, a minor role [70-73].

In addition to the intracellular mechanisms of toxicity described above, results from numerous studies have demonstrated that inflammation may enhance APAP-induced liver injury [74,75]. The earliest evidence for a contribution of inflame-mation to APAP hepatotoxicity was the finding that resident macrophages in the liver (Kupffer cells) are activated after APAP overdose in rats [76] and that inhibition of macrophages with gadolinium chloride was protective in that model [77]. The latter finding was later repeated in mice [78]. Similarly, it was also reported that antibodies against neutrophils can protect against APAP hepatotoxicity in rats and mice [79,80]. Finally, damage-associated molecular patterns (DAMPs) are released during APAP hepatotoxicity in both mice and humans [35,36] and several studies revealed that inhibition of Nalp3 inflammasome-mediated DAMP signaling in myeloid cells can reduce the injury [81-84]. However, the conclusions from those studies are controversial. Gadolinium chloride has numerous effects other than macrophage inactivation that could also explain protection against hepatotoxicity, and it was reported that targeting macrophages with liposomal clodrinate actually exacerbated the APAP-induced liver injury [85]. Furthermore, deficiency of Nalp3 signaling components does not protect against APAP toxicity, and modulation of IL-1β signaling also has no effect [86,87]. For more detailed information about sterile inflammation in APAP hepatotoxicity, the reader is directed to two excellent reviews that have recently been published [74,75].

Importantly, it appears that the mechanisms of APAP hepatotoxicity are the same in both humans and mice. Both GSH depletion [88,89] and APAP-protein binding are known to occur in humans [27,90] and oxidative stress, Jnk 1/2 activation and the MPT have been demonstrated in human hepatocytes treated with APAP [50,73]. Finally, there is evidence that mitochondrial damage is important in human APAP hepatotoxicity too [35,36,91].

## 3 Nephrotoxicity

*Evidence.* Numerous studies have shown that large doses of APAP can cause kidney injury in rodent models [4,15,92-96] and many reports of kidney injury in humans after APAP overdose have been published [3,97-102]. An often-cited figure for the overall incidence of renal dysfunction in patients diagnosed with APAP overdose is approximately 1%. However, this was derived from a single early review of unselected patients diagnosed with “APAP poisoning” at a single center in the UK [103]. Multiple reports suggest that the prevalence of renal injury among APAP overdose patients who develop liver injury is much greater; values from 10% to 79% have been reported [98,99,102-105]. One study found that circulating creatinine levels were ≥ 2 mg/dL (177 µmol/l) (reference interval: 0.7-1.2 mg/dL or 60–115 µmol/l) in approximately 50% of APAP-induced ALF patients, and the levels were higher in non-survivors compared to survivors [105]. Those data were supported by later studies that showed plasma creatinine level at admission and serum kidney injury molecule 1 (KIM-1) are predictive of poor patient outcome after APAP overdose [98,106]. Interestingly, some evidence suggests that chronic use of low doses of APAP can increase risk for kidney disease and cause analgesic nephropathy [107,108], although that has been questioned by findings from very large studies of “healthy” individuals who regularly use over-the-counter analgesics including APAP [108].

*Proposed mechanisms.* Although the nephrotoxicity of APAP has been known about for decades, surprisingly few studies have explored the mechanisms. Early on, it was thought that endotoxemia as a result of failure of the damaged liver to eliminate endotoxins from the normal GI flora was responsible for the renal damage [104], but results from later studies suggested a more direct effect involving reactive metabolites of APAP and APAP-protein binding [109]. There are significant species differences, and even within-species strain differences, in renal metabolism of APAP [110]. In Fischer F344 rats, APAP and NAPQI appear to be converted to p-aminophenol (PAP) by deacetylation in the kidney, and PAP can be further metabolized to a reactive quinone imine other than NAPQI, possibly by a prostaglandin endoperoxide synthase (PGES; aka cyclooxygenase, COX) [110-115]. Based on those data, it was initially thought that APAP nephrotoxicity was mediated by PAP. However, it was later demonstrated that inhibition of deacetylation had no effect on covalent protein binding in renal microsomes from Sprague-Dawley (SD) rats [116], and an antibody against the N-acetyl moiety of APAP-cysteine could bind to APAP-protein adducts in the kidneys of mice after APAP treatment but not after treatment with p-aminophenol [117]. Furthermore, covalent binding in renal microsomes from SD rats can be prevented by the P450 inhibitor 1-aminobenzotriazole [116], and the nephrotoxicity of APAP in mice is reduced by the P450 inhibitor piperonylbutoxide [117]. It is also apparent that sex differences in APAP nephrotoxicity in mice are due to differences in renal P450s. Female mice are resistant to renal injury even at doses of APAP that cause hepatotoxicity, and that is likely due to hormone-induced differences in P450 expression. Castration of male mice reduces APAP metabolism and protects against APAP-induced kidney injury [118], while testosterone injections induce Cyp2e1 and render female mice susceptible to APAP nephrotoxicity [119]. Together, those data strongly suggest that APAP nephrotoxicity in mice is mediated at least in part by P450s and the same reactive metabolite of APAP that causes liver injury. Which species (mouse or rat) and which strain (F344 or SD rats) is more relevant for human APAP nephrotoxicity is not yet known. PAP and PAP metabolites have been detected in urine from humans after APAP ingestion [120,121], which may suggest that deacetylation of APAP to PAP occurs in humans. However, PAP and APAP metabolism are difficult to disentangle. Furthermore, we know that the mouse is a better model for the liver injury caused by APAP [39]. Aside from cytochrome P450s, results from studies using isolated rabbit and human kidney microsomes have indicated that a PGES/COX can also convert APAP to NAPQI (via a phenoxy radical intermediate) [122]. Interestingly, more recent studies showed that renal injury after APAP overdose in mice is exacerbated by free APAP-cysteine from APAP-GSH [95,96]. APAP-cysteine generated from the breakdown of APAP-GSH in the GI tract and kidneys can act as an acceptor of the γ-glutamyl moiety of GSH in the GSH cycle, and thereby exacerbate GSH depletion in the kidneys [96].

Overall, it appears that NAPQI formation and protein binding are critical, similar to the liver. There is also some evidence that APAP can inhibit mitochondrial respiration in kidney cells from rodents [123,124]. However, little is currently known about APAP nephrotoxicity beyond those results. Although it is tempting to assume that the mechanisms are the same as in the liver due to the involvement of protein binding and mitochondria, there is currently no direct evidence for oxidative stress, kinase activation, or the MPT in APAP nephrotoxicity.

*Biological relevance of proposed mechanisms.* Nephrotoxicity is clearly a risk after APAP overdose. Available data suggest that protein binding and mitochondrial dysfunction occur in the liver after APAP overdose and that the injury is exacerbated by glutathione cycling, but much more work is needed to prove the importance of those phenomena in APAP nephrotoxicity. This is especially important because acute kidney injury is a predictor of poor patient outcome after APAP overdose [99,106], possibly because it contributes to death after APAP overdose through multi-organ failure. The high affinity of the PGES for APAP has prompted some to speculate that it is responsible for the increased risk of kidney disease after chronic low-dose exposure to the drug [122,110,115], but again, the occurrence of APAP nephrotoxicity among therapeutic users is controversial. We recommend that future research on APAP nephrotoxicity be focused on the importance of mitochondrial dysfunction and kinase signaling and treatments that could address those, as well as mechanisms of renal cell recovery that have been demonstrated to be important in other models of acute kidney injury [125].

## 4 Pulmonary toxicity

*Evidence.* There is evidence for a link between chronic APAP exposure at therapeutic doses and respiratory disease. A survey of general practice clinic patients in the UK found a positive association between frequency of APAP use and signs of asthma [20]. The same group also found that regional sales of acetaminophen in Europe correlated with incidence of respiratory illnesses [126] and that prenatal exposure to APAP may be associated with asthma, wheezing and other respiratory problems later in life [127]. Since then, other groups have obtained similar findings [128-130]. APAP exposure has also been associated with development of chronic obstructive pulmonary disease [131]. However, the conclusions from these studies are controversial. Several possible confounding factors have been suggested [132-134]. Among these, indication bias (“reverse causation”) is probably of greatest concern. For example, children with respiratory infections are more likely to be exposed to APAP as a part of normal treatment [135], which may lead to a false association between APAP exposure early in life and later asthma when in fact the later respiratory problems may be a result of the infection or related issues. There is some evidence of pulmonary toxicity in rodent models. Bronchiolar epithelium necrosis has been observed in mice treated with very large doses of APAP [15,16,136], but those data are clearly not relevant for the chronic low-dose exposures that are thought by some to cause asthma and other lung diseases. There is some evidence that low doses of APAP are proinflammatory in the lungs [17]. Furthermore, adult mice that were exposed to APAP in utero were found to have a greater response to an allergic challenge later in life [18]. However, additional work is needed to understand the pathophysiological significance of the latter phenomena. Overall, there is currently a tentative link between APAP and pulmonary disease that requires further investigation.

*Proposed mechanisms.* It has been suggested that chronic exposure to APAP can deplete GSH in the lungs and that this could explain a connection between APAP and respiratory diseases if it enhances susceptibility to oxidants, such as reactive-oxygen species produced by inflammatory cells or even environmental oxidants [20]. GSH depletion and increased expression of oxidative stress response genes have been detected in lungs from mice treated with large, acutely toxic doses of APAP and that could suggest oxidative stress [137-139]. APAP-protein binding in the lung has also been demonstrated in mice [137,140-142]. In fact, one study found that a polymorphism in glutathione-s-transferase (GST) P1 that reduces its activity was associated with wheeze in children exposed to APAP prenatally [129], although a conflicting study reported that wheezing and asthma in children of mothers who used APAP during pregnancy is greater when the mother possesses multiple copies of GSTP1 and/or GSTM1 compared with null genotypes [139].

A more specific mechanism of APAP-induced lung disease that has been proposed is neurogenic inflammation. Nassini et al. [17] suggested that inflammation develops in the lungs after APAP treatment due to activation of the transient receptor potential ankyrin 1 (TRPA1) channel in peptidergic neurons by NAPQI. They demonstrated that direct treatment with NAPQI can enhance Ca2+ uptake in cells expressing TRPA1. Importantly, there was also evidence for increased TRPA1 signaling and evidence of inflammation in lungs from rodents treated intratracheally with NAPQI or either intragastrically or intraperitoneally with relatively low doses of APAP (15-300 mg/kg). The authors were even able to detect sulfhydryl adducts after the 15 mg/kg dose, though it’s not clear what effect this had on total GSH levels or if protein binding actually occurred.

*Biological relevance and future studies.* Although GSH depletion has been demonstrated in lungs from mice overdosed with APAP, it is not clear if that occurs after repeated exposure to APAP at therapeutic doses, which would be more relevant for the reported epidemiological connections between APAP and chronic lung disease. Moreover, the GSH depletion that has been observed in lung is unimpressive: only about 30% of total lung GSH is lost even after treatment with a dose as large as 500 mg/kg [137]. It is possible that the GSH depletion selectively occurs in certain cell types in the lungs (e.g. Clara cells), in which case the total GSH would not be expected to dramatically change; however, covalent protein binding also has not been observed except at very high doses [137,140-142]. The TRPA1 hypothesis has more data to support its biological relevance. Unfortunately, the authors of that study used multiple models, including cultured cells, rat liver slices, isolated guinea pig trachea and mice to perform different experiments in the same study [17], and it’s not clear how each model is related. Furthermore, there was no assessment of pulmonary function in an in vivo model treated with APAP, so the physiological consequences of the inflammation are unknown. The authors did, however, test the effect of APAP on pulmonary insufflation pressure in vivo in guinea pigs and reported no change [17]. Thus, the evidence for TRPA1-mediated lung damage in animals is preliminary and should be further explored. Overall, it is not yet clear if or how APAP causes lung disease. We recommend that experiments measuring GSH and protein binding in the lungs be repeated in mice using low, therapeutic doses to determine if those mechanisms are actually relevant for humans. Presently, the most compelling data suggest that NAPQI can activate TRPA1 on neurons and lead to neurogenic airway inflammation, but a more detailed study using only the mouse model, and that includes assessment of pulmonary function, is needed to test that.

## 5 Endocrine disruption and sexual development

*Evidence.* It is critical to evaluate claims regarding long-term effects of intrauterine APAP exposure because APAP is currently the only drug recommended for pregnant women to reduce pain and fever. Modestly increased risk of cryptorchidism after prenatal exposure to APAP has been reported in humans in a few studies [143-145], which suggests some estrogenic or anti-androgen activity of APAP. However, the results are inconsistent and difficult to interpret together. For example, one study examined two patient cohorts and discovered an effect in only one of them [145]. Another study found that the risk of cryptorchidism was increased in offspring of mothers who used APAP for ≥ 4 weeks during pregnancy, but the likelihood of the child undergoing orchiopexy (surgical treatment, and therefore a surrogate marker of long-term cryptorchidism) was not [144]. Another study failed to find an association between APAP alone and other measures of androgen exposure, such as penis width and anogenital distance (AGD), commonly associated with reproductive disorders, despite an association with APAP and NSAIDs together [146]. Overall, there does not seem to be a clear relationship between APAP exposure during development and reproductive effects in humans. Nevertheless, several studies using rodent models have indicated a connection. One group has reported that intrauterine APAP exposure modestly affects AGD in male and female rodents [145,147,148] and may affect germ cell proliferation in female mice [148]. However, although they claimed to use subtoxic doses, the authors treated the animals with 50-350 mg/kg of APAP every morning for 7 days. While the maximum recommended dose of APAP in humans is approximately 50-60 mg/kg/day, that amount is typically divided into multiple smaller doses over a 24 h period. In fact, it is well known that a single treatment with ≥150 mg/kg is hepatotoxic in mice, resulting in significantly elevated plasma ALT values and evidence of hepatocyte necrosis by histology [149]. It is not surprising that there may be developmental abnormalities in offspring of animals that suffer liver injury during pregnancy. In fact, the most surprising finding from these studies may be that the effects were not more pronounced. Adding confusion to the debate, the same group recently found that 50 mg/kg/day has no effect on masculine behaviors or morphology in a region of the brain associated with those behaviors in male offspring [150], though the 150 mg/kg/day dose did have an effect. Overall, there is currently no clear association between APAP and reproductive effects in offspring.

*Proposed mechanisms.* APAP does not seem to be directly estrogenic [151], so other mechanisms have been proposed. One possible mechanism for the suggested endocrine-disrupting effects of APAP is altered sex steroid metabolism. Interestingly, one research group obtained moderately elevated values for total estrogen metabolites in urine from premenopausal women who reported high APAP use [152]. The only rodent in vivo study to address this issue revealed that plasma testosterone decreased after APAP treatment in castrated mice with human testis xenografts, which suggests that APAP decreases testosterone production in human testes [153]. Finally, a few in vitro studies have demonstrated that cytochrome P450-mediated steroid metabolism can be altered by APAP [154,155], though other studies have provided partially conflicting results [156]. Treatment of an adrenocortical carcinoma cell line resulted in increased pregnenolone and decreased androstenedione and testosterone in two studies [144,157]. Estrone and β-estradiol were also increased by APAP [147]. However, another study found no effect of APAP on testosterone production in human fetal testis [156]. Another mechanism that has been proposed for the possible endocrine-disrupting effects of APAP is reduced prostaglandin synthesis due to cyclooxygenase inhibition. Certain prostaglandin levels have been shown to decrease in cultured human fetal testis after APAP treatment [156].

*Biological relevance and future studies.* Altogether, there are limited and conflicting results regarding the endocrine effects of APAP. There is some epidemiological evidence for modestly increased risk of indirect markers of abnormal sexual development after intrauterine exposure to APAP in humans, but those data are by no means conclusive. Although one human study reported increased urine estrogen in humans after APAP use [152], it is unlikely that the modest effect that was observed would have a major impact on development. Even the evidence for developmental effects of prenatal use of potent, direct estrogens like oral contraceptives on sexual development in offspring is weak at best [157]. While results from some studies using cell culture models do support an effect of APAP on hormone metabolism, others have revealed conflicting results. Moreover, most of those studies involved prolonged treatment (24-72 h) with µM to mM concentrations of APAP, which is not consistent with the pharmacokinetics of APAP in vivo. Finally, the data from the human testis xenograft model are compelling, but the human relevance of that model is unclear. Overall, there is currently no strong evidence that intrauterine exposure to APAP can significantly alter sexual development or reproductive health later in life. Before any further research on the endocrine and reproductive effects of APAP or the mechanisms involved, we recommend that a simple study be performed in which pregnant mice receive a low dose of APAP (15 mg/kg) one to four times per day for several days and multiple developmental parameters of offspring health, including AGD and other measurements of reproductive health, is assessed. That will also require an evidence-based consensus on what are the most important or relevant reproductive health parameters to measure.

## 6 Ototoxicity

*Evidence.* At least 19 reports of rapidly progressive sensorineural hearing loss caused by abuse of APAP/opioid combinations have been published [158-160]. In most cases, the hearing loss is bilateral, suggesting a systemic cause consistent with drug exposure. In vitro studies have demonstrated that long-term (≥24 h) exposure to high concentrations (mM) of APAP can reduce the number of viable cells in isolated cochlea (particularly in the outer hair cells) and cause evidence of apoptotic cell death in an auditory cell line (HEI-OC1) that was derived from the organ of Corti in the ImmortomouseTM model [161] and is generally thought to represent cochlear hair cells [3,8]. Interestingly, co-treatment with hydromorphone enhanced APAP ototoxicity in these models, though hydromor-phone or hydrocodone alone did not cause cell death [8]. NAPQI was shown to have similar effects [13]. Those data suggested that APAP is the primary cause of hearing loss due to APAP/opioid abuse. However, no clinical reports of hearing loss after overdose of APAP alone have been published. Furthermore, the same group published a more recent study indicating that APAP does not actually cause cell death in HEIOC1 cells, despite evidence of reduced energy metabolism and even increased caspase activity [162]. Finally, a recent in vivo study in mice found no evidence for hearing loss based on auditory brainstem response (ABR) in a clinically relevant model of acute APAP overdose [163]. Thus, it seems unlikely that APAP by itself causes ototoxicity in humans or mice. Nevertheless, a practical clinical problem clearly exists in patients treated with opioid/APAP combinations and further investigation may be warranted.

*Proposed mechanisms.* Kalinec et al. [13] found that APAP can cause evidence of oxidative stress in HEI-OC1 cells 12-48 h after initiation of treatment, but that NAPQI does not have this effect. Furthermore, increased endoplasmic reticulum (ER) fragmentation was observed in these cells after treatment with NAPQI but not APAP [13]. Despite the latter, both treatments altered levels of ER stress markers. Based on these findings, the authors concluded that APAP and NAPQI exert toxic effects through different mechanisms in cochlear cells: APAP ototoxicity involves oxidative stress and ER stress, while NAPQI causes ER stress without oxidative stress [13]. The only in vivo study of APAP ototoxicity to date also revealed that there is oxidative stress in cochleae after acute APAP overdose [163]; however, no ototoxicity was observed in that study based on auditory brainstem thresholds (ABR) [163].

*Biological relevance and future studies.* While interesting, the results from cell culture studies thus far are questionable. First, APAP has a very short half-life in circulation [26]. Thus, it is unlikely to persist at the cochlea for ≥ 24 h, as in the in vitro experiments described above. Although some drugs (e.g. aminoglycosides) may become trapped within the cochlear fluid, this is unlikely to occur with APAP because it is neutral at physiological pH and readily crosses membranes [26]. Next, it is not known if HEI-CO1 cells, or cochlear cells in general, express P450s at concentrations sufficient to convert APAP to NAPQI. The only study to address that issue revealed that mice treated with a hepatotoxic dose of APAP had no evidence of GSH depletion or protein binding in cochlea [163]. Finally, it is clear that APAP toxicity in vitro does not necessarily translate to toxicity in vivo. Many cell lines succumb to APAP toxicity through mechanisms that are not physiologically relevant. For example, both Hepa 1-6 and SK-Hep1 liver cells will die after prolonged exposure to mM concentrations of APAP, despite the fact that these cells do not form the reactive metabolite of APAP [164 , 165]. Importantly, the primary mode of cell death in these cells was found to be apoptosis, which is not a major contributor to APAP-induced hepatocyte death in vivo [35,50,70, 166]. Furthermore, APAP is also toxic to human lymphocytes in culture [165], but there is little or no evidence that that is true in vivo. Clearly, it is important to realize that cell culture studies do not necessarily mimic the in vivo situation. Overall, it is clear that APAP/opioid combinations are ototoxic in humans, but there is no strong evidence that APAP is ototoxic by itself. Future research in this area is encouraged, and should focus on hearing loss caused by the combination drugs, and should use only in vivo models with clear human relevance.

## 7 Neurodevelopmental and neurobehavioral disorders

*Evidence.* Several groups have claimed that APAP may be a cause of autism spectrum disorder (ASD) [7,11,14]. Two major pieces of evidence led to that hypothesis. First, it was observed that at least some patients with ASD exhibit defective xenobiotic sulfation [167]. In fact, when APAP was used as a probe drug to assess sulfation capacity, the ratio of APAP-sulfate to APAP-glucuronide was lower in severely autistic subjects compared to healthy controls [176]. Initially, it was suggested that this could lead to poor clearance of, and therefore increased exposure to, certain chemicals present in food or in the environment that may have neurological effects, but it was later proposed that APAP itself might be a problem. Schultz et al. [7] suggested that reduced sulfation may lead to increased NAPQI formation with neurotoxic effects. Second, it was found that diagnoses of ASD began to increase in the 1980s, after the CDC issued a warning regarding the risk of Reye’s syndrome and birth defects when treating children or pregnant women with aspirin, and sales of children’s APAP rose [168]. However, it is unlikely that reduced sulfation would lead to a significant increase in NAPQI formation at therapeutic doses of APAP. Sulfation is a low capacity route of elimination and is already saturated in healthy subjects at pharmacologic doses of APAP [169]. Glucuronidation, on the other hand, is a high capacity process and does not appear to be saturable [27]. In fact, the hepatotoxicity of APAP is probably not due to saturation of Phase II metabolism resulting in greater NAPQI formation; the percentage of APAP converted to the reactive metabolite is likely the same regardless of dose. Rather, it is probably the greater absolute amount of NAPQI that is produced that initiates liver injury after overdose [27]. Furthermore, the observed correlation between children’s APAP sales and ASD diagnoses does not prove causation.

Nevertheless, several groups have reported results from epidemiological studies that seem to show an association between APAP exposure early in life and development of ASD [7,11,170]. One of the earliest such studies revealed that parents of children with autism were more likely to report use of APAP after receiving the measles-mumps-rubella vaccine [7]. However, it has been pointed out by others that the parents were solicited from autism websites and thus were likely to be biased [171]. In addition, there is the possibility of recall bias in parents of children with autism who are in search of a cause [171]. More recent studies have employed more rigorous methods [170]. Unfortunately, even those that have marginalized the risk of indication bias may still be affected by genetic factors or other residual bias [172]. Overall, the only human data available to support the idea that APAP causes ASD are from epidemiological studies that may be subject to significant bias.

In addition to ASD, it has recently been suggested that antenatal exposure to APAP may cause hyperactivity or ADD / ADHD-like behavior in offspring. Liew et al. [7] found an association between APAP and these disorders in a large prospective cohort study, and their results are supported by data from a few other groups [173-176]. However, significant sources of bias have been pointed out in three of these studies as well [177], and earlier work by Streissguth et al. [178] provided conflicting results. Interestingly, one group has even tested the association between prenatal APAP exposure and ADD/ADHD-like behavior in mice and found no evidence to support it [179], although it should be noted that there were clear experimental deficiencies such as a lack of well-validated endpoints for ADD/ADHD in mice and the fact that a positive control is not available for comparison. Overall, there is currently no strong evidence that APAP causes ADD/ADHD.

Although the evidence for neurobehavioral effects of APAP in humans is poor, multiple studies have demonstrated that exposure to relatively low doses of APAP during early development can affect behavioral measures in adult mice [12,180]. While it is not possible to make a direct connection between non-specific behavioral studies in mice and ASD or ADD/ADHD in humans, these observations are intriguing and may warrant further investigation. Typically, pregnant women are advised not to use NSAIDs due to the increased risk of birth defects and miscarriage that has been reported in a few studies. As a result, most pregnant women rely on APAP to control fever and pain. If it can be shown that APAP also poses a significant risk of congenital abnormalities, then that may result in removal of the only remaining treatment option for those patients.

*Proposed mechanisms.* The proposed mechanisms by which APAP could cause ASD and ADD/ADHD are similar. Endocrine disruption, activation of endocannabinoid receptors during development [181], oxidative stress and inflammation [182] have all been suggested. However, no studies have been done to directly test those possibilities. A more straightforward hypothesis is that APAP is directly toxic to neurons. Posadas et al. [9] tested that by treating rat cortical neurons with APAP in vitro and by injecting rats with APAP in vivo and measuring neuron death. They demonstrated that APAP overdose was moderately toxic to cortical neurons. However, the purpose of their study was to determine if large doses of APAP (250-500 mg/kg) are neurotoxic, and it is not known if typical human doses for therapeutic use (approximately 10-20 mg/kg) have similar effects. Cell death in APAP-treated cultured neurons has also been reported [9], but again most cell culture models do not accurately reflect APAP toxicity in vivo. Finally, it is not clear exactly how neuron death would lead to ASD and ADD/ADHD.

*Biological relevance and future studies.* Currently, the association between APAP and ASD or ADD/ADHD is based on conflicting results from epidemiological studies. No mechanistic studies have been performed, and the few mechanisms that have been proposed have not been directly tested. In fact, there is strong evidence that ASD, in particular, is driven by genetics [183], so exposure to APAP or other xenobiotics may not be important. Males are far more likely to develop ASD, and siblings of children with ASD are at greater risk [183]. There is also striking evidence for a genetic component of social behaviors associated with ASD, such as viewing of social scenes [184]. Nevertheless, the importance of APAP as a treatment option during pregnancy, together with the seriousness of ASD and ADD/ADHD, warrants future research in this area to enable more definitive conclusions. Even a simple study could be performed in which pregnant mice receive 15 mg/kg APAP one to four times per day for several days and behaviors associated with ASD and ADD/ADHD are measured in offspring over time.

## 8 APAP toxicity in other tissues or systems

APAP toxicity has been reported in other tissues, but the evidence is limited. For example, APAP is also known to cause ocular opacity or cataracts in mice, but only after direct induction of P450 enzymes in ocular tissue [185,186]. It has also been suggested that APAP can be cardiotoxic, but this is based on case reports with no direct evidence [187]. Currently, there is no compelling evidence for clinically-relevant APAP toxicity in tissues other than those discussed above.

## 9 Conclusions

It has been 50 years since the first reports of APAP-induced liver injury, and we are only beginning to investigate the extrahepatic toxicity of the drug in earnest. Renal toxicity after APAP overdose is known to occur, but the mechanisms have not been fully elucidated. It is also not known if common comorbidities like alcoholism or obesity affect that outcome. The pulmonary and neuro- toxicity of APAP are more controversial. Most data regarding the non-hepatic and non-renal effects of APAP are from epidemiological studies that do not prove causation and frequently suffer from bias and/or conflicting results. Published experimental data provide support for many of these adverse effects, but too often the data come from flawed models. However, we believe that some additional research may be appropriate in at least two areas. The sheer volume of epidemiological studies that have revealed increased risk of lung disease after exposure to APAP early in life and the fact that at least one group has reported a plausible mechanism based on data from animal models using low doses of APAP may warrant further investigation of the pulmonary toxicity of chronic APAP use. Also, the fact that APAP is a very important drug for pregnant women combined with the several rodent studies suggesting adverse neurodevelopmental effects in offspring may warrant further investigation of neurodevelopmental toxicity to fully evaluate that possibility. Overall, however, the data for extrahepatic toxicity of APAP are weak and significant changes in clinical or consumer use would be not advisable at this time.
